# Laser patterned, high-power graphene paper resistor with dual temperature coefficient of resistance†

**DOI:** 10.1039/c8ra10246e

**Published:** 2019-03-12

**Authors:** Sandeep Kumar, Kapil Bhatt, Pramod Kumar, Sandeep Sharma, Amit Kumar, C. C. Tripathi

**Affiliations:** RF & Flexible Microelectronics Research Laboratory, Department of ECE, University Institute of Engineering and Technology, Kurukshetra University Kurukshetra-136119 India kapilbhattuiet@gmail.com; Amity Institute of Advanced Research and Studies (Materials and Devices), Amity University Noida-201313 India

## Abstract

Printing of electronic devices on a paper substrate using 2D graphene-based ink is an opening gate to innovative applications, where devices would be biodegradable, eco-friendly and can be disposed of with negligible impact on the environment. A resistor is a key element of electronic devices and their application area depends upon its power rating and temperature coefficient of resistance (TCR). In this work, in house developed graphene ink is successfully utilized to fabricate a paper-based resistor using a bar coating technique. Dimensional patterning with precise known values of resistance is achieved using a laser with freedom of shape and size which has been explored for the first time on a paper substrate. The resistor has potential to handle ∼7 W power at room temperature with capacity to withstand up to 200 V which is the highest among reported printed resistors. A dual, low and high TCR is observed, correspondingly in cold (173 K to 300 K) and hot (300 K to 373 K) temperature regions with an activation energy *E*_a_ of ∼8 meV for the cold region which is 375 percent lower than the hot region (∼30 meV). The dual TCR behaviour is of great importance for application as a stable resistor up to room temperature, and as a thermistor above room temperature.

## Abstract 2

Printing of electronic devices on a paper substrate using 2D graphene-based ink is an opening gate to innovative applications, where devices would be biodegradable, eco-friendly and can be disposed of with negligible impact on the environment.

## Graphical abstract

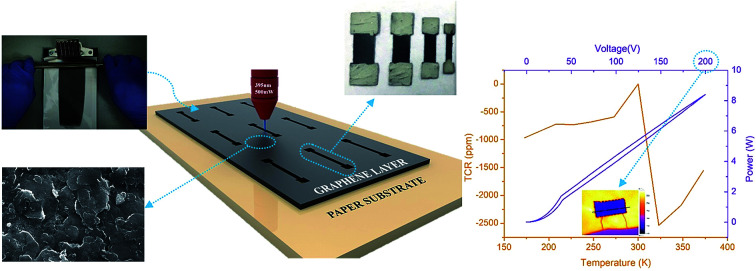

## 1. Introduction

In recent years, the demand for small, lightweight, flexible and thin film devices for applications such as consumer electronics, defense, health monitoring, aerospace engineering and many more is increasing day by day which motivates researchers to work in the field of flexible and printable electronics.^1,2^ Flexible printed electronic devices can be fabricated on a variety of substrates waving off the restrictions of silicon with add-on advantages like low cost, roll-to-roll process,^3^ large area printing^4^*etc.* Printing technologies like inkjet, screen, and flexography are widely in use for the production of functional structures on an arbitrary substrate such as polymers and paper.^3^ But their utilization is restricted due to the inconsistent range of ink parameters such as rheology, particle size, surface tension^5^*etc.* and optimization of these parameters is a very tedious job as a small change in one deviates others significantly. Comparatively, the bar coating technique can be successfully implemented for a broader range of ink parameters. Various nanoparticles such as copper, silver, and carbon nanotubes are utilized to formulate functional inks and used to fabricate flexible printed devices. Graphene has advantages over the traditional nanoparticles, due to its tremendous properties like higher mechanical strength, excellent flexibility, transparency, and higher conductivity. The exceptional flexible properties of graphene are by virtue of its crystalline structure in which sp^2^ hybridized carbon atoms connect with each other and the high electrical conductivity is due to the formation of π-bonds. These properties made graphene a strong contender for the fabrication of flexible devices using printing technology.^6–12^ Graphene has also been explored for the fabrication of various active and passive flexible devices like Field Effect Transistor (FET),^13^ antenna,^14^ super-capacitor,^15^ resistor,^16^ sensor^17^*etc.*

On the basis of the resistor electrical resistance property, this two terminal passive electrical component is ubiquitous in various electronic circuits. Presently, resistors are being fabricated using various novel methods as compared to their earlier fabrication procedures. Now a days screen printing,^18^ vacuum filtration & cutting,^19^ chemical vapor deposition,^20^ and ink jet printing^3,16^*etc.* are a few techniques used to fabricate resistors. Ink formulation is a customized job which is defined by the printing process, substrate, layer thickness, drying mechanism *etc.* Therefore, the in-house developed graphene ink for the fabrication of resistors for flexible electronics systems is explored. The temperature coefficient of resistance (TCR) is an important parameter of a resistor, to be used as a temperature sensor or as a stable passive component. The TCR value and its nature are two defining parameters of a device which decides whether it can be used as a temperature sensor or as a stable resistor in high-reliability circuits and systems.^21^ More emphatically, in the case of temperature sensing applications, the device is supposed to show high TCR, on the other hand a resistor for reliable circuits must have a very low TCR.^22^ The TCR of the printed resistor mainly depends upon the temperature coefficients of the ingredients of the ink along with the humidity and pressure of the surrounding environment. It has been reported that humidity, temperature and other gases present in the environment affect the properties of graphene and consequently affect the resistance and TCR.^23–27^ There are numerous studies on the TCR of graphene-based resistors on various substrates. However, the nature of the TCR reported in the literature is contradictory *i.e.* in some cases the value of the TCR reported is near zero or very low and other cases very high.^16,28–31^ This makes it necessary to explore the value of the TCR in graphene-based resistors in detail. In this work, resistors were fabricated on a paper substrate using graphene ink with bar coating film deposition technique and laser cutting to result in a predetermined resistance value. Further detailed study to understand the mechanism for the observed high/low value of the TCRs is presented. In this work, the dual TCR nature of the graphene-based resistor on paper substrate is presented for the first time to the best of our knowledge.

## 2. Results and discussion

Homogeneously dispersed graphene ink was formulated with the help of a vibroshaker using a combination of organic solvents to get proper wettability, stability and a fissure-free homogenous printed layer (the detailed formulation procedure is described in the Materials and methods section). The combination of solvents enhances dispersion of the solid components and substrate wettability along with smoothness in the printed layer. Solvents get evaporated after thermal treatment and therefore do not impact the conductivity. Other additives such as resin, sticking agent, wetting and dispersion modifier which remain in the coated layer contribute to an increase in resistance due to their insulating nature.^32^ Graphene was purchased for the ink formulation and in order to analyze the quality, Raman spectroscopy of the printed resistor from formulated graphene ink was carried out (Fig. S1 (curve ‘a’), ESI†). The D and G bands which are the salient Raman modes to be present are clearly observable near 1355 and 1589 cm^−1^ respectively. The G band originates from the stretching of the covalent bond in the carbon lattice whereas the D band gives a signature of the present disorder in the structure. Further, the 2D band observed at 2729 cm^−1^ is an overtone of the D band signifying the formation of the multilayer structure of the graphene ink printed resistor. For better comparison, the Raman spectrum of the purchased graphene powder was also done at the same excitation wavelength and laser power (Fig. S1 (curve ‘b’), ESI†). From this overlay we observed the broadening in the 2D band of the printed ink as compared to that of powder graphene; moreover both are related to the multi-layered structure of graphene (∼5 layers).^33^ Such broadening may be attributed to the stack formation during printing of the ink. However, the ratio of the intensity of the D band w.r.t. that of the G band depicts the quality of the graphene-like structure. For powdered graphene the ratio *I*_D_/*I*_G_ has been found to be 0.16 which increases to 0.23 in the printed sample; still this ratio is quite low thus confirming the formation of a good quality graphene ink printed resistor.^34,35^

**Fig. 1:**
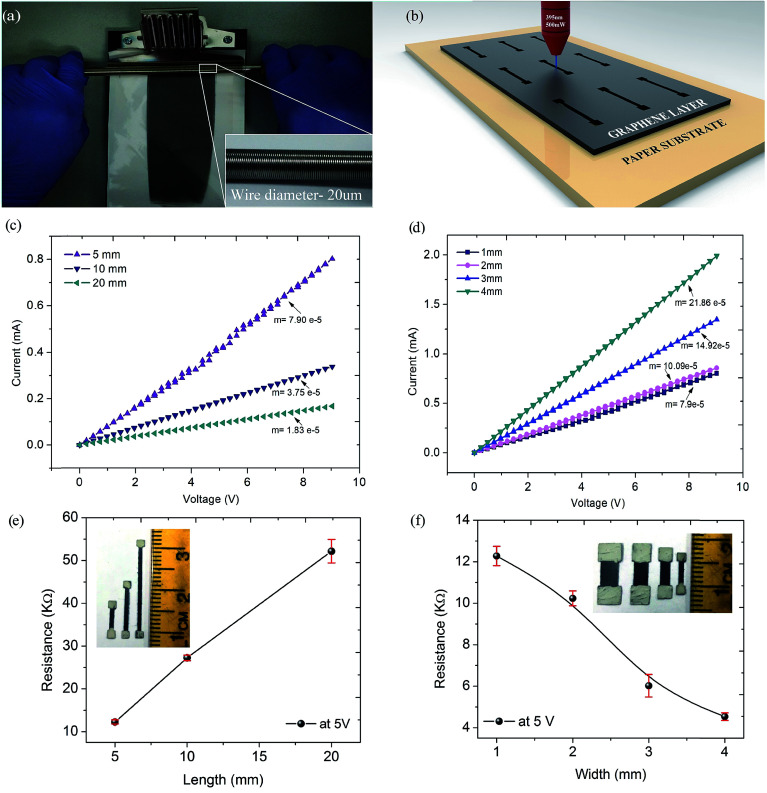
(a) Bar coating of graphene ink on paper substrate. (b) Resistor patterned by laser. (c) *V*(*I*) characteristics of the graphene resistors with width of 1 mm and lengths 5, 10, 20 mm; (d) length of 5 mm and widths 1, 2, 3, 4 mm. Effect of resistor’s (e) length, (f) width on the resistance.

In the present work, graphene ink was primarily coated using a wire wound bar coater on the paper substrate (Fig. 1a). Predefined size paper resistors were trimmed out using a laser beam in the shape of dumbbells as shown in Fig. 1b (detailed device fabrication procedure is described in the Materials and methods section). Three resistors of lengths 5, 10, and 20 mm with width 1 mm (30 nos. ) and four resistors of widths 1, 2, 3, and 4 mm with length 5 mm (30 nos.) with a 2 × 2 mm contact area were used for the characterization. *V*(*I*) characteristics of the resistors were recorded and follow Ohm’s law. Variation in the curve’s slope indicates that the current decreases apropos of voltage with an increase in length and increases with an increase in width as shown in Fig. 1c and d. Resistors having certain resistance value can be customized by selecting dimensional parameters as per Pouillet’s law. Fig. 1e and f confirm that mean resistance of the graphene film is a linear function of its length and width which clearly follows Pouillet’s law. Error bars in the resistance values are very small and easily acceptable for the commercial circuits.^36^ Through selecting these dimensional parameters graphene film resistors with customized resistance values can be fabricated roll-to-roll using a laser source.

**Fig. 2:**
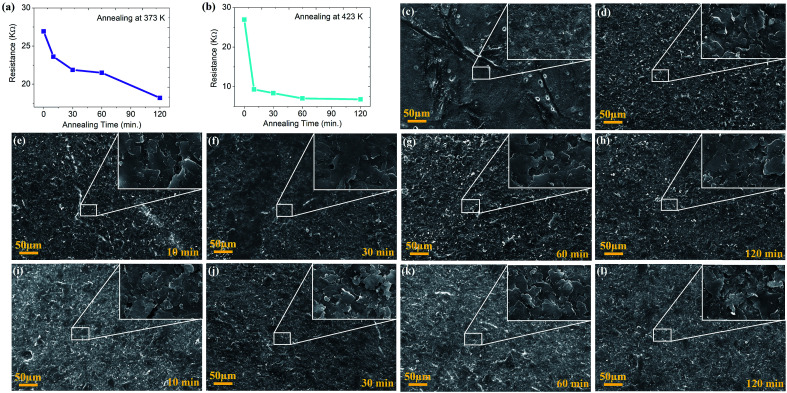
Change in resistance of the device w.r.t. annealing time at annealing temperature (a) 373 K and (b) 423 K. SEM images at 500× magnification (in inset at 10k× magnification) of (c) paper substrate, (d) graphene ink coated on paper substrate without annealing, (e–h) device surface after annealing at 373 K and (i–l) 423 K for 10, 30, 60, 120 min, respectively.

Therefore, this rapid method can be easily commercialized with the added advantage of below micron level dimensional precision with freedom of shape and size. Further, the function of annealing temperature for varying time on the morphological and electronic transfer characteristics of printed resistors has been explored in detail. Two annealing temperatures 373 K and 423 K for four distinct times (10, 30, 60, 120 min) have been selected to examine their effect on device performance. The measured resistance reduced from 27 kΩ to 23.6, 21.8, 21.5 for successive increases in annealing time and reaches the lowest of 18.5 kΩ for annealing time 120 min at 373 K (Fig. 2a). The reduction rate in resistance was 331 Ω min^−1^ when the sample was annealed for 10 min and drops to 13 Ω min^−1^ for the annealing time 60 min (Table S1†). At 423 K resistance drops a lot, from 27 kΩ to 9.3, 8.4, 7.1 for successive increases in annealing time and reaches the lowest of 6.8 kΩ for annealing time 120 min (Fig. 2b). The slope of resistance reduction in this case was very high (1761 Ω min^−1^), more than the sample annealed at 373 K for 10 min. After that it nearly stabilized with much less change (4 Ω min^−1^) as compared to the first 10 min. Solvents used in the ink boiled around 373 K and most of the volatile organic components (VOCs) were evaporated around it. Thermogravimetric analysis (TGA) of the sample also discloses this and after that there is a nearly stable mass of ink up to 650 K (Fig. S2†).

As the annealing time increases, it impacts less because most of the solvent was evaporated in the first 10 min and nothing much has been left to fly away. TGA analysis of the graphene ink coated paper also supports this conviction (Fig. S3†). However, morphological study of the top surfaces using SEM did not show any significant changes because of the similar morphology of cellulose (paper substrate) and graphene as shown in Fig. 2c and d. This morphological similarity limits the measurable changes in the surface and means that while annealing rearranging of the graphene layers may happen leading to a decrease in resistance of the coated layer (Fig. 2e–l).

The temperature–time profile of the samples on annealing demonstrated that the annealing time has less effect on device resistance than the annealing temperature. Therefore, more annealing time can be traded for less annealing time, which requires the annealing temperature must increase to drive-off excessive remnant VOCs. Reduction in annealing time is advantageous to expand the portfolio of materials that would otherwise be excluded in roll-to-roll device fabrication on flexible substrates. Longer annealing time is not acceptable in roll-to-roll printing of electronic devices which significantly affects the process’s throughput. The annealing temperature is still low and easily acceptable for polymer substrates in flexible and printable electronics with the added advantage of low annealing time.

**Fig. 3:**
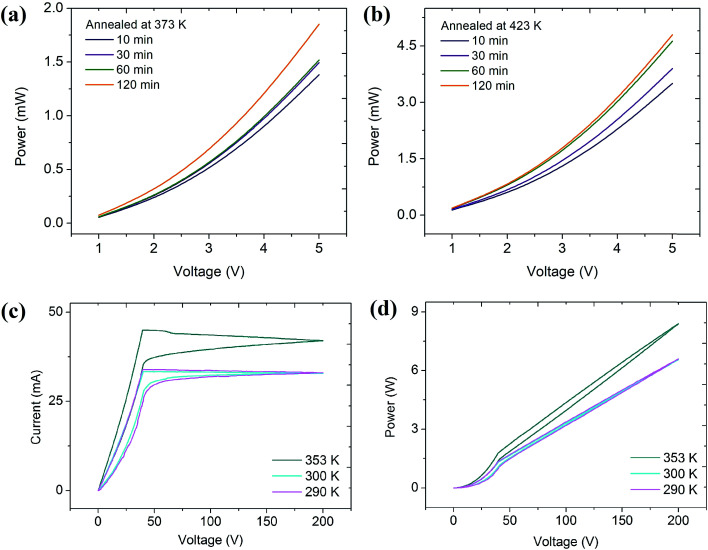
Output power spectra (up to 5 V) for the resistor after annealing at temperature (a) 373 K, (b) 423 K and (c) *V*(*I*) characteristics and (d) power spectra (up to 200 V at 290 K, 300 K and 353 K) of resistor annealed at 423 K for 120 min.

SEM analysis of the as fabricated resistor reveals the uniformity in micro-structure of the material. While a lower resistance value with high temperature bearing capability leads the design of a resistive, high power structure on paper substrate and exhibited high performance characteristics which will be described here in detail. High power resistors have applications as resistance heaters, in current sensing, snubber applications, bleeder resistor, inrush current limiting, ballast resistor, electronic dissipater and so on.^37–39^ The operational power of the printed devices was explored with diverse annealing times and temperatures to examine their power operational capabilities as well as the best parameters which can deliver maximum power. Fig. 3 shows output power variations of samples that were annealed at 373 K (a) and 423 K (b), for 10 min to 120 min. Power was measured up to 5 V for all annealed samples (sample dimensions = 5 × 1 mm).

The maximum power measured was 5 mW at 300 K for the sample annealed for 120 min at 423 K. Then the potential bearing capability of the device with temperature variance was further evaluated using an in-house developed cryostat (Fig. S4†). The lowest valued resistance (*l* × *w* = 5 × 4 mm) for that purpose was taken into account which hopes to convey the highest power by keeping in mind that power is inversely proportional to the resistance. The sample was then annealed for 120 min at 423 K. The potential difference up to 200 V was applied and the power delivered by the device measured was 6.5, 6.6 and 8.4 W at temperature 290 K, 300 K and 353 K respectively (Fig. 3c and d). Reproducibility of the devices was tested by performing repeated cycles at different temperatures and similar responses have been observed every time. During the electronic transport characteristics measurement of the device it was found that the device exhibited linear pure ohmic behavior between voltage and current up to 40 V (Fig. 3c) after that the current approaches towards saturation and reaches to its maximum value of 33, 37 and 42 mA at 200 V at temperature 290 K, 300 K and 353 K respectively. This change in current with respect to temperature indicates the negative temperature coefficient of resistance of the device.

While tracing back the measurements, current was constant up to 40 V and below that it started decreasing in the case of all three measurements. Hysteresis loss has been observed in this study and was very low 0.5, 0.4 and 0.8 Joules per cycle for temperatures 290 K, 300 K and 353 K respectively, which is quite acceptable. Ohmic behaviour (up to 40 V) in the *V*(*I*) characteristics (Fig. 3c) clearly indicates the semiconductor behavior of the devices. Beyond 40 V, charge transport may be limited by saturation of the number of charge carriers resulting in non-ohmic behavior. Besides this current saturation, power delivered by the fabricated device was nearly similar to the previously reported inkjet printed resistor^16^ with added advantages of bearing a very high potential of more than 200 V. The stability of the device at such high voltage was tested using IR (infrared) thermal imaging and it was found that there was no increase in temperature after operating the device for 60 seconds at 200 V (Fig. S5†). Paper as a substrate strongly reflects its opportunity to be used in roll-to-roll fabrication of electronic devices for biodegradable electronics. There is a huge burden of aged electronic devices to the environment causing pollution and health hazards to living beings. So, it is necessary to develop devices which will affect the environment to a minimal level after completing their life cycle. That’s why paper as the substrate and graphene as the conducting material are a nice combination to fabricate future devices which will be flexible as well as biodegradable in nature with significant performance.

**Fig. 4:**
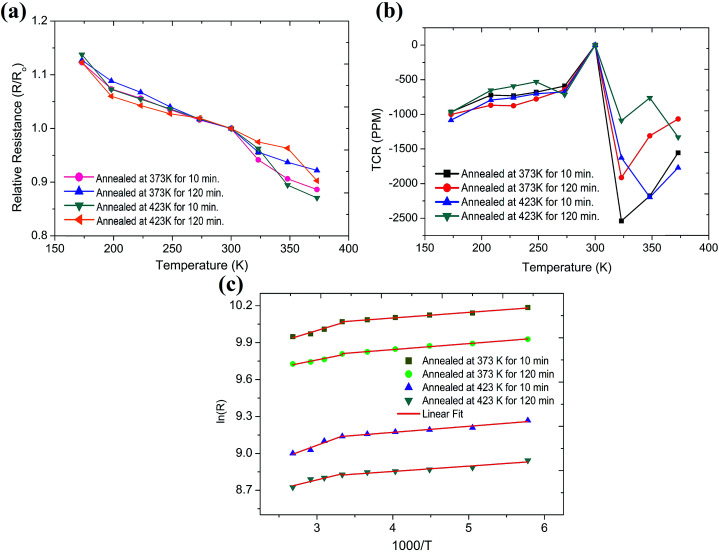
(a) Relative resistance and (b) TCR of graphene paper resistors at varied annealing temperature and time; (c) logarithmic plot of ln(*R*) as a function of 1000/*T* with linear fitting.

In order to better understand the electronic transport characteristics of the laser patterned multilayer graphene resistors, *R*–*T* measurements of the devices were carried out from 173 to 373 K and their TCR, thermal index and activation energy were calculated. The changes in resistance have been studied in cold and hot regions taking room temperature (300 K) as the reference temperature. For the measurement of the TCR the following equation is used1
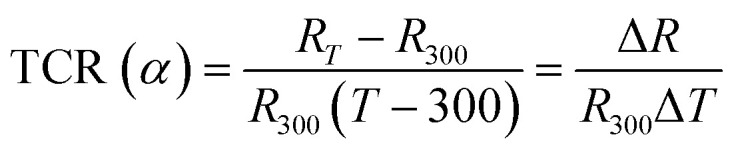
where *α* is the temperature coefficient of resistance of the device, Δ*R* is the change in resistance w.r.t. change in temperature (Δ*T*) and *R*_300_ is the resistance of the device at 300 K. Fig. 4a shows the deviations in relative resistance with respect to temperature variations. Commercially, TCR is measured in PPM °C^−1^ or PPM K^−1^ by multiplying *α* by 10^6^. Previously reported graphene resistors have shown a high TCR and have been used as temperature sensors while some have shown a near zero TCR which can be used as a stable resistor.^28–30^ It is the intrinsic behaviour of the synthesized graphene material and defects occurring during synthesis that are the crucial factors which are responsible for low or high observed TCR.^28^ Here dual TCR behavior of the device is being reported which can be used as a stable resistor along with as a temperature sensor, as shown in Fig. 4b. The variation in resistance below room temperature is quite insignificant therefore the resistor can be considered as a stable resistor, however, at temperatures above room-temperature, the resistor shows high resistance variance indicating its ability to behave like a temperature sensor. Generally, any such major change in electrical conductivity with temperature variance is expected due to phase transition due to deformation and reorganization of the conductive network.^40^ Therefore, to verify any change in phase, differential scanning calorimetry (DSC) of the graphene, resin and ink was carried out, but no evidence of phase transition was found (Fig. S6†). Consequently, this phenomenon may occur due to an increase in carrier concentration with temperature as observed in the case of another semiconductor.^41^

The semiconductor behavior with temperature is divided into two regions, *i.e.* extrinsic and intrinsic. The temperature at which this transition of regions occurs depends upon energy bandgap, charge carrier, defects and doping level.^29^ In this regard, the transition of region in the case of silicon occurs around 500 K, but in the present case the transition occurs at room temperature due to the lower bandgap. However, monolayer graphene has zero bandgap but doping and increasing the number of layers could tune this.^42^ Eda *et al.* also reported a similar type of TCR transition of reduced graphene oxide by various means and their impact on TCR.^43^ It was found that annealing temperature and time have a strong influence over TCR. The sample annealed at 373 K has a higher TCR value than at 423 K, as well as annealing time playing a vital impact on TCR which decreases with increase in annealing time.

The variance in resistance with temperature can be further used to extract the thermal index (*B*) and activation energy (*E*_a_) of the fabricated resistor by using the Arrhenius equation mentioned below:

2 *R* = *R*_0_e^*E*_a_/2*kT*^ = *R*_0_e^(*B*/*T*)^

where *R* is the resistance at temperature *T*, *R*_0_ is the resistance at infinite temperature (*T*) = ∞, *B* is the thermal index (Fig. 4c), *k* is the Boltzmann constant and *E*_a_ is the thermal activation energy. From the above equation *E*_a_ can be obtained using the below mentioned relationship:

3 *E*_a_ = 2*kB*

By using these equations the values of TCR, thermal index and activation energy were calculated and summarized in Table S7.† The negative (−ve) TCR of the fabricated devices for all conditions confirms the semiconductor behaviour of the graphene. The slope of the TCR curve also confirms that the change in electrical resistance is dominated by graphene rather than insulating resin and other additives.^44^ There is a huge difference in TCR (∼216%) of the cold and hot zones for all studied conditions (Table S2†) which confirms that dual TCR is the inherent property of the formulated graphene ink. This increase in TCR will become greater if we further proceed our measurements towards higher temperatures. Perusal of the data presented in ESI Table S2† reveals the value of the goodness of fit parameter (Adj. *R*^2^) is from 0.86 to 0.99, indicating a reasonable linear fitting of experimental data. The measurement conditions played an important role and therefore, above room temperature measurements were done in both vacuum and a normal environment at relative humidity of about 50%. Humidity has a direct influence on graphene resistance and it has been confirmed by researchers previously.^25,45^ Along with this, the paper substrate has a hygroscopic nature therefore temperature and humidity both are responsible for the high value of TCR in the normal environment (Fig. S7 and S8†). Despite this, the mean value of the thermal index in the hot region was found to be 174, which is 375 percent higher than the cold region when measured in vacuum (Table S2†). Further, the TCR, *E*_a_, *B* and the working temperature range of the fabricated resistors are compared with the reported devices on rigid and flexible substrates and are summarized in Table 1.

**Table 1:** Comparison table of high, low and near zero (NZ) TCR materials on various substrates specifying thermal index, activation energy, and TCR values along with the measurement temperature range from previous reports

Temperature response	Material	Substrate	Thermal index, *B* (K)	Activation energy, *E*_a_ (meV)	TCR PPM K^−1^	Temperature range	Reference
High TCR	Graphene	SiO_2_/Si	1034	177.84	−11480	300 to 353 K	30
High TCR	Graphene	PDMS	946	167.2	−10500	303 to 373 K	28
**High TCR**	**Graphene**	**Paper**	**170 ± 40**	**30 ± 7**	**−1770 ± 480**	**300 to 373 K**	**Present work**
**Low TCR**	**Graphene**	**Paper**	**50 ± 3**	**8 ± 1**	**−800 ± 50**	**173 to 300 K**	**Present work**
Low TCR	Printed multilayer graphene film	Polyimide and PET	14	2.4	−150	6 to 350 K	16
NZ-TCR	Carbon nanotube + carbon black	PDMS	5	0.86	−50	300 to 473 K	29
NZ-TCR	Antiperovskite Mn_3_Ni_1–*x*_Cu_*x*_N	Antiperovskite Mn_3_Ni_1–*x*_Cu_*x*_N	2	0.34	−20	10 to 360 K	31
NZ-TCR	Graphene–rGO	Silicon	0.003	0.0006	0.23	303 to 373 K	45

Al-Mumen *et al.*^30^ reported a high value of TCR (−11 480) for a graphene-based device, however, these devices are on a SiO_2_/Si substrate and are not considered for flexible electronic devices. Similarly various low TCR resistors have been reported by other researchers, but most of them are on rigid substrates and can’t be considered for roll-to-roll fabrication of devices.^31–45^

## 3. Materials and methods

### Graphene ink formulation

Graphene powder was purchased from United Nanotech, India and other materials were borrowed from CYMK inks LLP, India. A vibroshaker (Darteno Industries, India) was used for graphene ink formulation with the following composition: graphene powder (10 grams), resin solution (40 grams), ANTI-TERA-U (0.5 grams), MEK (34.5 grams), methyl isobutyl ketone (10 grams), cyclohexene (5 grams), *n*-propyl acetate (5 grams). All constituents were pre-mixed in an SS container using an electric stirrer (Rescholar Devices, India) for 5 minutes. The graphene ink was then shaken by the vibroshaker using 100 grams of zirconium oxide beads (0.5 mm diameter) to act as grinding media for 2 hours in four intervals of half-hour each having a half-hour resting period between two successive shakings to avoid over heating of the ink. After shaking, the ink was separated from the zirconium oxide beads and stored in an air-tight glass bottle. The detailed characterization of the as formulated ink will be reported elsewhere.

### Thick graphene film fabrication

Thick graphene film of approximate thickness 20 μm was deposited using a wire wound bar coater (RK PrintCoat, UK) of thread size 20 micron on a paper substrate (RK Print Coat, UK) of thickness 71 ± 8 μm with surface roughness of 0.9 ± 0.4 μm.

### Graphene resistor fabrication

The resistors were first designed in Inkscape software (Open Source Vector Graphics Editor). The graphene coated film was patterned by laser (wavelength 395 nm, 500 mW power) to fabricate the resistor.

### Electrical characterization

Electrical contacts of the resistor to the external electrical measurement system were made by silver paste (borrowed from Indo Solar, India). A Keithley 2450 source meter was used to take the electrical measurements in vacuum with the help of a Cryostat. Low temperature measurements (173 to 300 K) were carried out in cryogenic conditions using liquid nitrogen as the coolant.

### Other characterization

Surface morphological characterization was carried out using a scanning electron microscope (SEM) on a Zeiss (EVO-18). Thermogravimetric analysis (TGA) was performed using a HITACHI STA 7200 in the presence of nitrogen gas (N_2_) and a heating rate of 10 K per minute was maintained during the characterization. Differential Scanning Calorimetry (DSC) measurements were carried out using an SDT Q600 analyzer (TA Instruments) in the presence of nitrogen gas (N_2_) and a heating rate of 2 K per minute was maintained during the characterization.

## 4. Conclusion

A high power and dual TCR device was successfully bar coated using stable graphene ink. Characterization of the film by electrical measurements showed that the optimum annealing temperature and time are 423 K and 10 min, respectively, which makes it suitable for roll-to-roll device fabrication. Laser patterned bar coated devices have been successfully fabricated and demonstrated high power handling capacity up to 7 W along with high voltage bearing capability of 200 V. The devices showed dual TCR behaviour in cold and hot regions separately which is believed to be the first report amongst previously studied 2D materials. It can be suitably used as a stable resistor in the low temperature region and as a thermistor at above room temperature.

## Conflicts of interest

All authors do not have any conflict of interest.

## Supplementary Material

RA-009-C8RA10246E-s001
