# Supplementary material for: Laser patterned, high-power graphene paper resistor with dual temperature coefficient of resistance
Source: RSC Adv. 2019 Mar 12;9(15):8262–70. doi: 10.1039/c8ra10246e (PMC9061268; doi:10.1039/c8ra10246e)
Supplement: RA-009-C8RA10246E-s001 [file RA-009-C8RA10246E-s001.pdf]

Electronic Supplementary Material (ESI) for RSC Advances.
This journal is © The Royal Society of Chemistry 2019

Supplementary Information

Laser Patterned, High-Power Graphene Paper Resistor with Dual Temperature Coefficient of Resistance

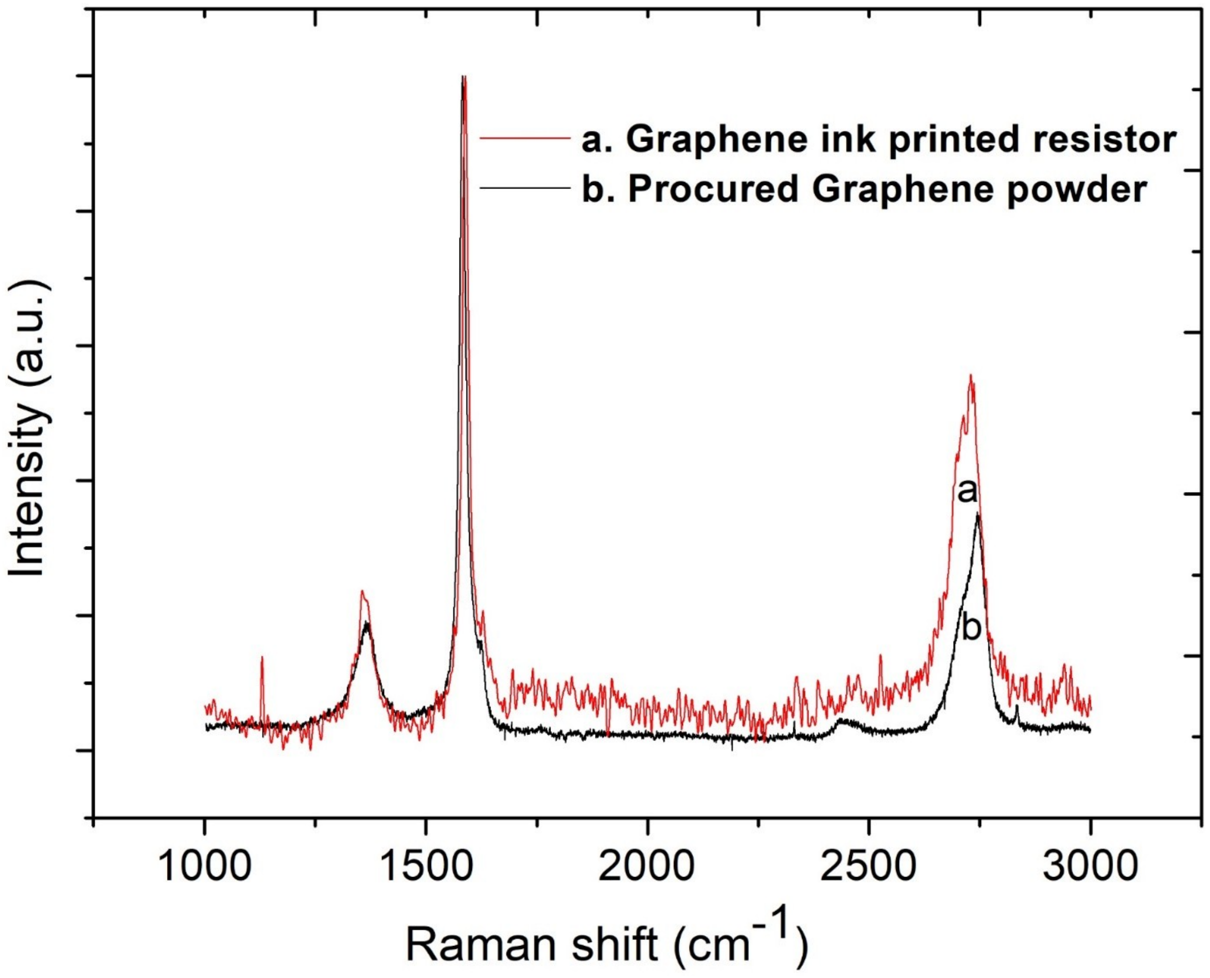

Figure S1: Raman spectra of Procured graphene powder and graphene ink printed resistor

Table S1: Change in resistance corresponding to the annealing temperature and time

Annealing Time (min.)	At 373K	At 423 K
	Change in Resistance per minute	
**10**	331	1761
**30**	88	47
**60**	13	44
**120**	55	4

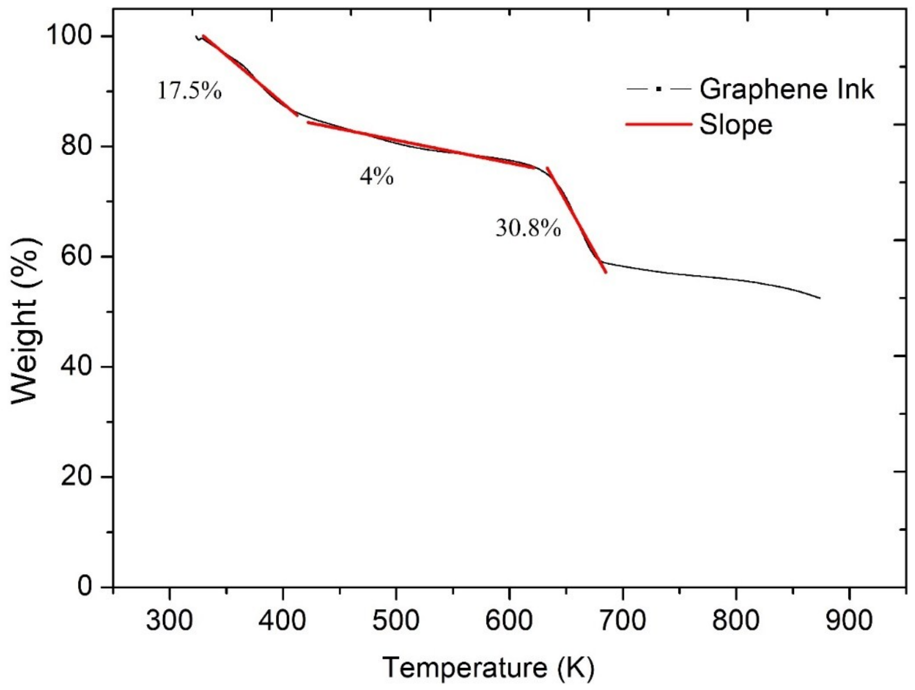

Figure S2: TGA of the graphene ink

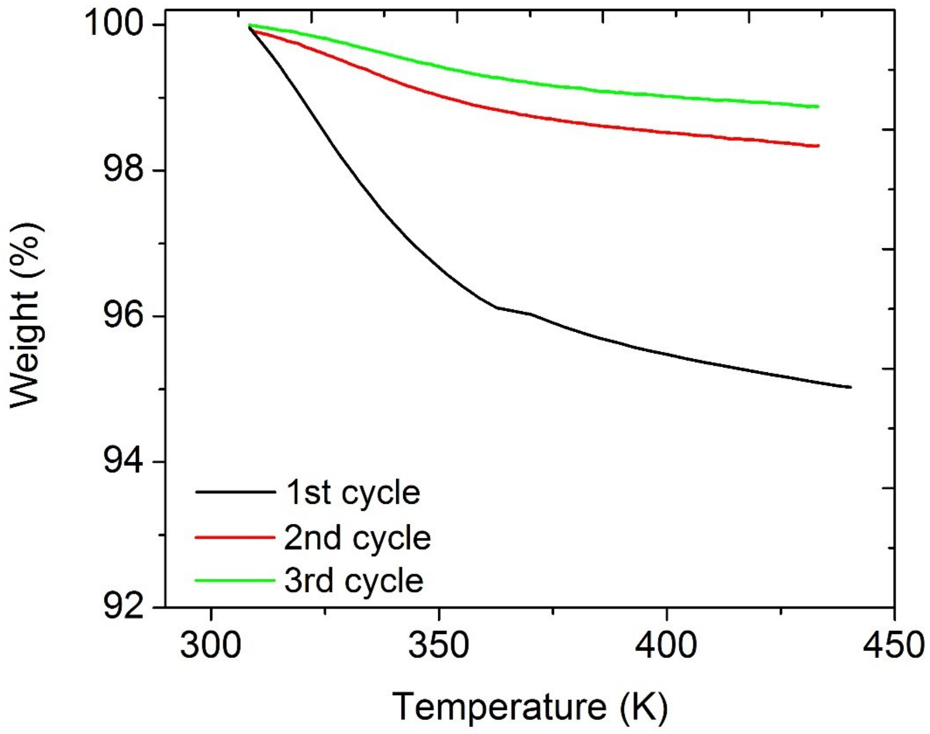

Figure S3: TGA of the graphene ink bar coated on paper

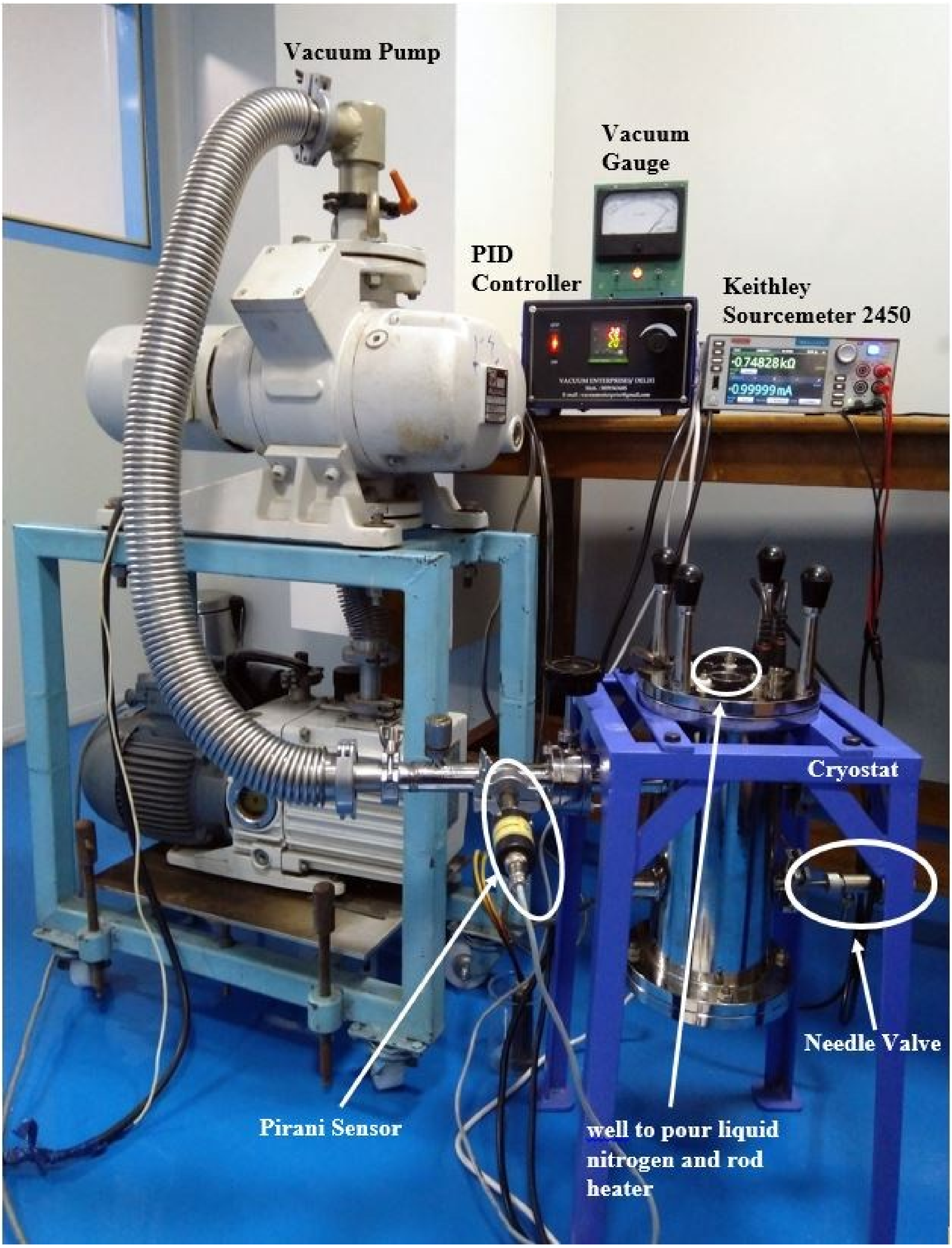

Figure S4: Cryostat setup for I-V characterization

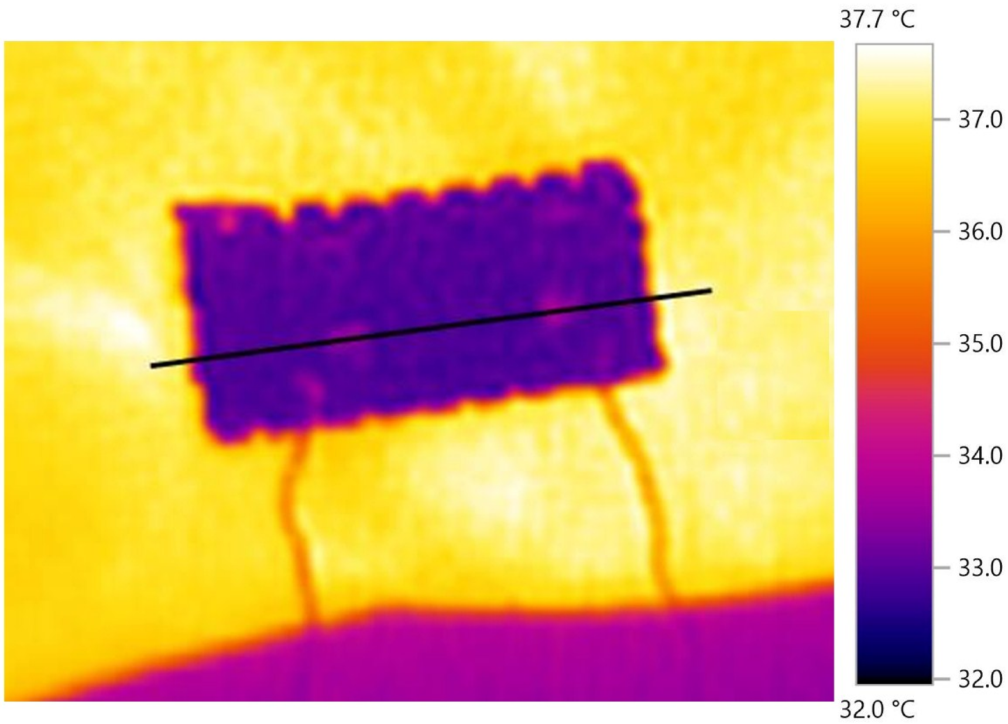

Figure S5: IR imaging of resistor after operating at 200V for 60 sec.

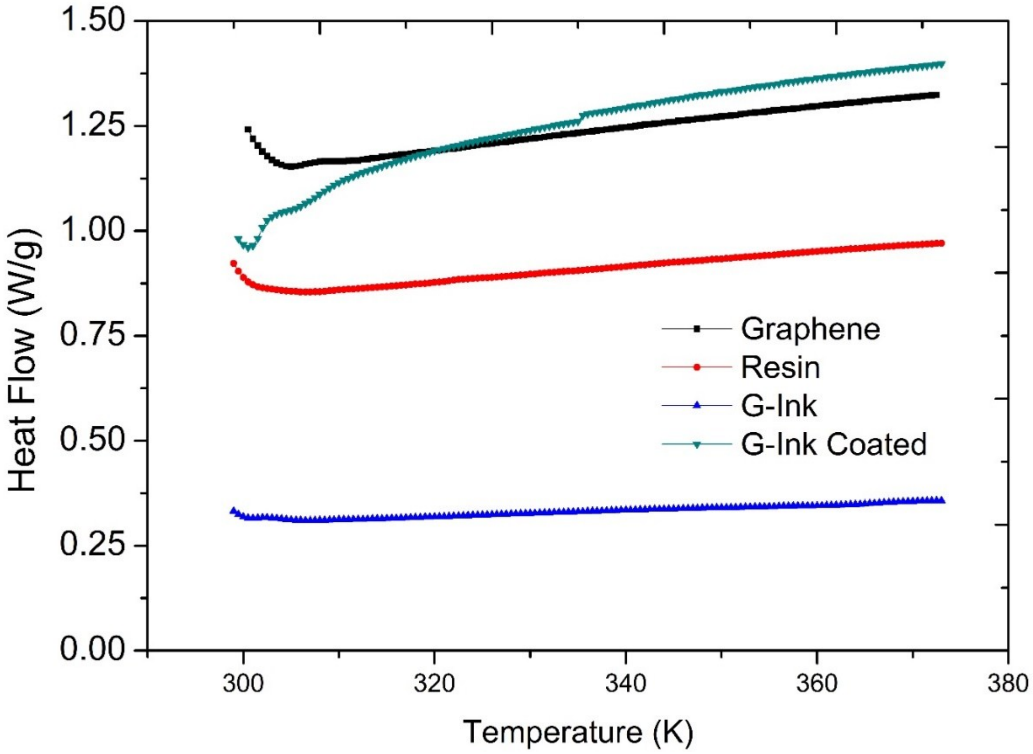

Figure S6: DSC profile of graphene, resin, graphene ink (G-Ink) and graphene ink coated on paper substrate

Table S2: Mean values along with standard deviation of TCR, thermal index and activation energy for variable annealing temperature and time

**Parameters**	**Annealed at 373 K for 10 min**		**Annealed at 373 K for 120 min**		**Annealed at 423 K for 10 min**		**Annealed at 423 K for 120 min**	
	**Cold Region**	**Hot Region**	**Cold Region**	**Hot Region**	**Cold Region**	**Hot Region**	**Cold Region**	**Hot Region**
**-ve TCR (PPM/K)**	750 ± 125	2100 ± 1000	850 ± 100	1480 ± 350	800 ± 150	2000 ± 400	700 ± 150	1100 ± 200
**Thermal Index (K)**	50 ± 10	190 ± 30	50 ± 2	125 ± 20	50 ± 5	230 ± 40	45 ± 6	150 ± 35
**Activation Energy (meV)**	8 ± 2	32 ± 5	9 ± 0.5	22 ± 4	9 ± 1	40 ± 6	8 ± 1	25 ± 6
**Adj. R-Square**	0.99	0.94	0.99	0.92	0.97	0.93	0.94	0.86

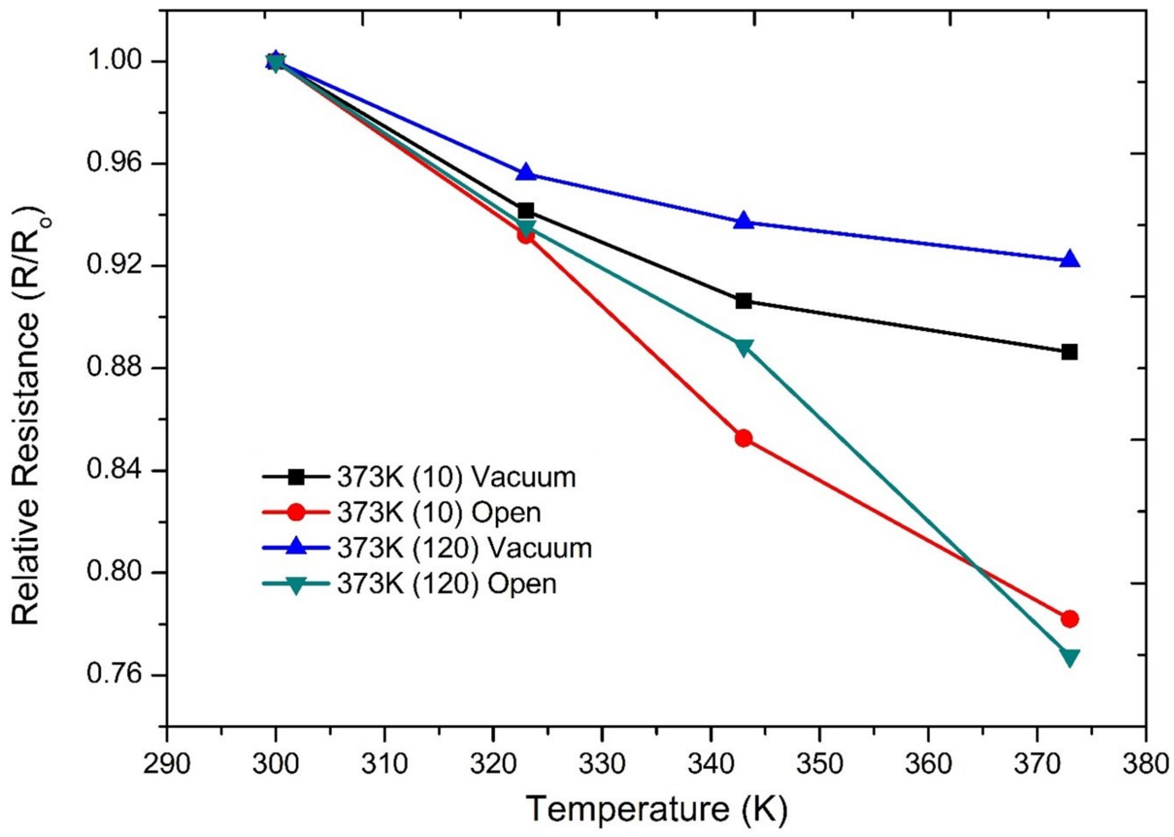

Figure S7: Change in relative resistance with temperature in vacuum and open for sample annealed at 373 K

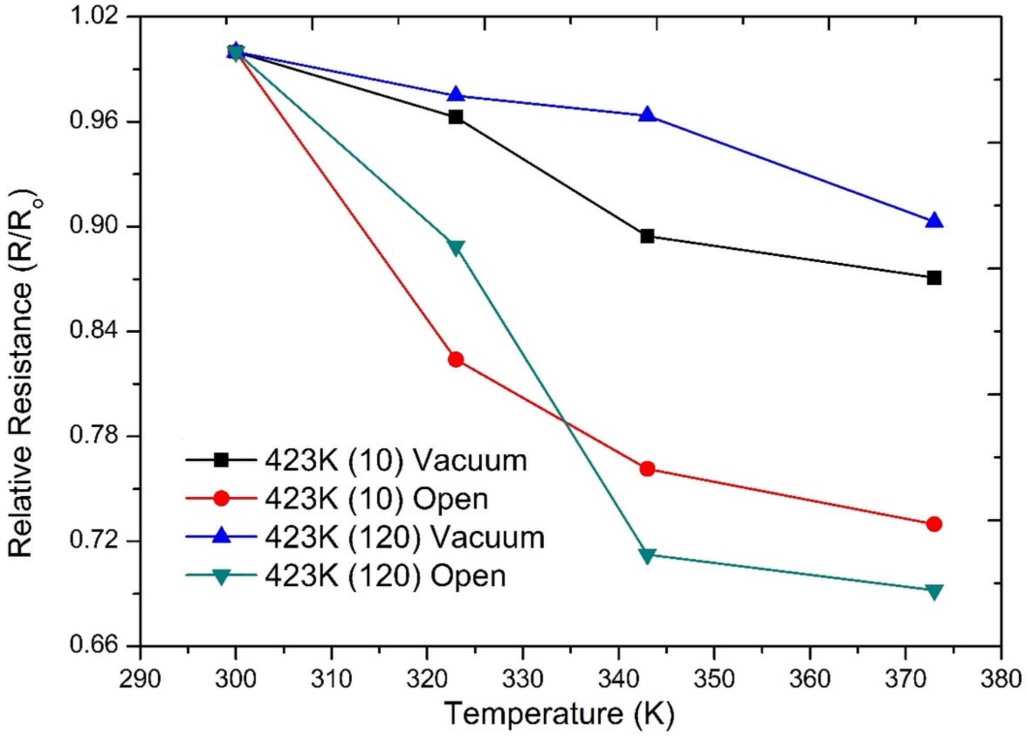

Figure S8: Change in relative resistance with temperature in vacuum and open for sample annealed at 423K
